# Nuclear Factor κB is Required for Tumor Growth Inhibition Mediated by Enavatuzumab (PDL192), a Humanized Monoclonal Antibody to TweakR

**DOI:** 10.3389/fimmu.2013.00505

**Published:** 2014-01-08

**Authors:** James W. Purcell, Han K. Kim, Sonia G. Tanlimco, Minhtam Doan, Melvin Fox, Peter Lambert, Debra T. Chao, Mien Sho, Keith E. Wilson, Gary C. Starling, Patricia A. Culp

**Affiliations:** ^1^Department of Biologics Technologies, AbbVie Biotherapeutics, Redwood City, CA, USA; ^2^Department of Oncology Biologics, AbbVie Biotherapeutics, Redwood City, CA, USA

**Keywords:** enavatuzumab, monoclonal antibody, TweakR, Fn14, NFκB, p21

## Abstract

TweakR is a TNF receptor family member, whose natural ligand is the multifunctional cytokine TWEAK. The growth inhibitory activity observed following TweakR stimulation in certain cancer cell lines and the overexpression of TweakR in many solid tumor types led to the development of enavatuzumab (PDL192), a humanized IgG1 monoclonal antibody to TweakR. The purpose of this study was to determine the mechanism of action of enavatuzumab’s tumor growth inhibition and to provide insight into the biology behind TweakR as a cancer therapeutic target. A panel of 105 cancer lines was treated with enavatuzumab *in vitro*; and 29 cell lines of varying solid tumor backgrounds had >25% growth inhibition in response to the antibody. Treatment of sensitive cell lines with enavatuzumab resulted in the *in vitro* and *in vivo* (xenograft) activation of both classical (p50, p65) and non-classical (p52, RelB) NFκB pathways. Using NFκB DNA binding functional ELISAs and microarray analysis, we observed increased activation of NFκB subunits and NFκB-regulated genes in sensitive cells over that observed in resistant cell lines. Inhibiting NFκB subunits (p50, p65, RelB, p52) and upstream kinases (IKK1, IKK2) with siRNA and chemical inhibitors consistently blocked enavatuzumab’s activity. Furthermore, enavatuzumab treatment resulted in NFκB-dependent reduction in cell division as seen by the activation of the cell cycle inhibitor p21 both *in vitro* and *in vivo*. The finding that NFκB drives the growth inhibitory activity of enavatuzumab suggests that targeting TweakR with enavatuzumab may represent a novel cancer treatment strategy.

## Introduction

TweakR (Fn14, TNFRSF12A) is a member of the TNF receptor superfamily which is activated by its ligand, the cytokine TWEAK (TNFSF12). TweakR is the smallest member of the TNFR superfamily (1). It lacks the death domain associated with other TNFR members such as TNFR1, Fas, and TRAIL-R1, but it does contain a cytoplasmic TNFR-associated factor (TRAF) binding site allowing recruitment of TRAF adapter proteins which are vital for many of the intracellular signaling events that occur downstream of the TNFR family (2, 3).

TweakR was initially described as an inducer of apoptosis in certain cancer cell lines upon stimulation with its ligand TWEAK (4). TweakR also has a role in diverse biological processes such as inflammation, tissue repair, angiogenesis, and cell migration (5–8). The signaling pathways downstream of TweakR have been elucidated for some of these biological functions. The ERK, JNK, and NFκB pathways have been shown to be upregulated by TWEAK in endothelial cells, while the NFκB pathway appears to be involved in TWEAK-stimulated inflammation and cell survival (9–11). TWEAK and agonist TweakR antibodies have also been shown to induce cell death in certain tumor cell lines through multiple mechanisms, including caspase-dependent and -independent apoptosis and necrosis (3, 12–14). However, in other tumor cell lines, TweakR stimulation leads to a slowed growth effect, not cell death (15). The signaling pathways mediating that phenotype have not been determined.

The ability of TweakR stimulation to inhibit the growth of certain cancer cell lines, as well as the observation that TweakR is over-expressed in many cancers (3, 16) suggested that an antibody targeting TweakR could be a potential therapeutic agent. Enavatuzumab (formerly PDL192), a humanized monoclonal IgG1, which binds to and activates TweakR, has been shown to have growth inhibitory activity in multiple solid cancer models both *in vitro* and *in vivo* and has been evaluated in a Phase 1 study (15, 17). In preclinical studies, the *in vivo* activity of enavatuzumab was attributed to both direct stimulation of TweakR and Fc-mediated antigen dependent cellular cytotoxicity (ADCC). The mechanism of how enavatuzumab directly inhibited the growth of tumor cells, and the cell signaling events occurring downstream of enavatuzumab binding to TweakR were undefined, and therefore became the primary focus of this study.

In this report we show that enavatuzumab activates the NFκB pathway, and that its growth inhibitory activity is dependent on NFκB. The finding that NFκB activation induced by the TweakR pathway drives the growth inhibitory activity of enavatuzumab provides an interesting function for the NFκB family which is more frequently associated with growth and survival of cancer cells than their inhibition (18, 19).

## Materials and Methods

### Antibodies and reagents

Enavatuzumab (PDL192), 19.2.1, and the human IgG1 control used in this study have been described previously (17). The enavatuzumab-Fc mutant contains the L234A, L235A mutations that reduce FcγR binding and ADCC. PDL400 (human IgG1) is a humanized version of the previously described ITEM-4 (13). Mouse anti-TweakR antibodies 136.1 (IgG1) and 18.3.3 (IgG2a) were generated using the same strategy as that described for 19.2.1 (17). Antibodies were used at 10 μg/mL for *in vitro* studies, unless otherwise stated, and crosslinked with F(ab′)_2_ goat anti-human IgG (Fc_γ_ specific) from Jackson ImmunoResearch at 3.5 μg/mL. Recombinant human TWEAK was purchased from R&D Systems.

siRNAs were purchased from Thermo Scientific Dharmacon and included the following: non-targeting control siRNA pool (D-001810-10-05), p65 (RelA) siRNA pool (L-003533-00-0002) and set of four individual siRNAs (LQ-003533-00-0002), p52/p100 (NFκB2) siRNA pool (L-003918-00-0002) and set of four individual siRNAs (LQ-003918-00-0002), p50/p105 (NFκB1) siRNA pool (L-003520-00-0002) and set of four individual siRNAs (LQ-003520-00-0002), RelB siRNA pool (L-004767-00-0002) and set of four individual siRNAs (LQ-004767-00-0002), IKKα (CHUK) siRNA pool (L-003473-00-0002) and set of four individual siRNAs (LQ-003473-00-0002), IKKβ siRNA pool (L-003503-00-0002) and set of four individual siRNAs (LQ-003503-00-0002), p21 (CDKN1A) set of four individual siRNAs (LQ-003471-00-0002).

### Cell lines

All cell lines were obtained from the American Tissue Culture Collection (ATCC) or National Cancer Institute (NCI), except HSC-3 which was purchased from the Japan Health Science Foundation and the MB231 variant cell line, which was derived from the MDA-MB-231 cell line for its increased metastatic potential *in vivo*.

### Cell viability assay

The 105 cell line panel was cultured at 500 cells per well in triplicate with enavatuzumab or IgG1 control in the presence of F(ab′)_2_ goat anti-human IgG (Fc_γ_ specific) for 5 days in 96 well plates. Relative cell viability was determined using CellTiter-Blue™(Promega). Fluorescence emitted at 590 nm was used to calculate the growth effect relative to the IgG1 control antibody treatment. Each cell line was tested twice with the average growth inhibition reported.

### Luciferase transcriptional reporter assay

The Cancer 10-Pathway Reporter Luciferase Kit was purchased from SA Biosciences [CCA-101L (Plate Format)] and reverse transfected into cells using Lipofectamine 2000 (Invitrogen). Cells were treated with the indicated antibodies for 24 h following transfection. Cells were harvested an additional 24 h later and reporter activity was measured using the Dual-Luciferase Reporter Assay (Invitrogen).

### Western blot analyses

Whole cell protein lysates were generated using Cell Signaling protein lysis buffer containing protease inhibitors (Roche) and phosphatase inhibitor cocktail (Sigma), quantified using BCA reagent (Pierce), and protein expression was detected using ECL Plus Chemiluminescence kit (GE-Amersham). P-IκBα (Ser32/36) 5A5, p21 WAF1/CIP1 (DCS60), p65, p100/p52, p105/p50, and RelB antibodies were purchased from Cell Signaling. GAPDH antibodies were from Santa Cruz. Mouse and rabbit secondary antibodies were from GE-Amersham.

### NFκB transcription factor ELISA

TransAM™ NFκB family ELISA kit (Active Motif) was used to determine NFκB subunit DNA binding and functional activity in response to enavatuzumab treatment. Nuclear protein extracts were generated and the subunit ELISAs (p65, p50, RelB, p52) performed according to the manufacturer’s guidelines.

### Gene chip analysis

RNA was isolated and purified with RNeasy Micro kits (Qiagen) and analyzed using an Agilent 2100 Bioanalyzer RNA 6000. cDNA was prepared from the RNA template, and used for *in vitro* transcription in the presence of biotinylated nucleoside triphosphates. The biotinylated RNA targets were fragmented, and hybridized to a customized Affymetrix genechip platform (Eos Hu03plus) using standard Affymetrix protocols. Genechips were performed in duplicate and stained with streptavidin phycoerythrin (SAPE) and scanned on an Affymetrix GeneChip Scanner 3000. Raw data files were obtained after analysis of scanned images with GCOS (GeneChip Operating Software, Affymetrix). Gene chip expression data was generated according to previously described methods (20). The microarray data contained within this manuscript is registered in the Gene Expression Omnibus (http://www.ncbi.nlm.nih.gov/geo/) and can be accessed using the GEO submission GSE51934.

### siRNA transfections

OnTarget Plus pooled siRNA (15 nM) (Thermo Scientific) against the target gene of interest or non-targeting pool control siRNA were reverse transfected with Lipofectamine RNAiMax (Invitrogen) according to manufacturer’s protocol. A positive control siRNA for successful transfection was also included (Kinesin Spindle Protein, KSP). Target knockdown was maximally achieved 2 days post transfection. Cells were then treated with enavatuzumab or IgG1 control + crosslinking antibody for the time indicated. The relative viability of the cells was determined, and cells were also harvested for Western blot analyses.

### IKK16 NFκB inhibition

The IκB kinase (IKK) selective inhibitor IKK16 (Tocris Bioscience) was used at 160 nM (21). The IKK16 was added to cells either alone or 1 h prior to addition of enavatuzumab (10 μg/mL) + crosslinking antibody (3.5 μg/mL). Cell viability was determined 5 days post treatment.

### Cell division analyses

HT3 cells (30,000 cells per six-well) were siRNA transfected as described above. Two days post transfection, CellTrace™ violet (Invitrogen) was added to the cells at 5 μM for 30 min, cells were washed according to the manufacturer’s instructions, and treated with enavatuzumab or IgG1 control in the presence of crosslinking antibody for 5 days. Cells were trypsinized and then fixed in 2% paraformaldehyde. The amount of CellTrace™ dye within the cells was analyzed by flow cytometry using a violet laser (CyAn, Dako).

### Xenograft generation

ICR SCID mice (Taconic) were injected subcutaneously with 10^7^ cells in RPMI media. Mice were randomized into groups when the average tumor volume reached ~100 mm^3^. The animals were injected with 10 mg/kg enavatuzumab or IgG1 control three times per week (i.p.). Where p21 expression was to be analyzed by IHC, mice received only a single dose of antibody. Animals were sacrificed at the various times indicated post-dose. Tumors were harvested and flash frozen for protein or fixed in buffered formalin and paraffin embedded. All animal protocols and procedures were approved by the vivarium’s Institutional Animal Care and Use Committee consistent with The U.S. Public Health Service *Policy on Humane Care and Use of Laboratory Animals* (Office of Laboratory Animal Welfare, National Institutes of Health).

### Immunohistochemistry

Xenograft samples were formalin fixed and paraffin embedded. Tissue sections (5 μm) were cut, mounted on slides, deparaffinized and ethanol rehydrated. Antigen retrieval was performed using BORG Decloaker RTU (Biocare Medical). Primary antibodies were p21 mIgG1 (Dako M7202) and cytokeratin18 rIgG1 (Abcam). Secondary antibodies were AlexaFluor488 goat anti-rabbit and AlexaFluor594 goat anti-mouse (Invitrogen). Slides were mounted in Vector Lab DAPI mounting medium and imaged on a Zeiss Axioskop two fluorescent microscope. The number of p21-positive cells in response to enavatuzumab or IgG1 control was determined by counting three 40× magnification fields for each xenograft tested.

## Results

### Enavatuzumab displays broad growth inhibitory activity *in vitro*

**Figure 1:**
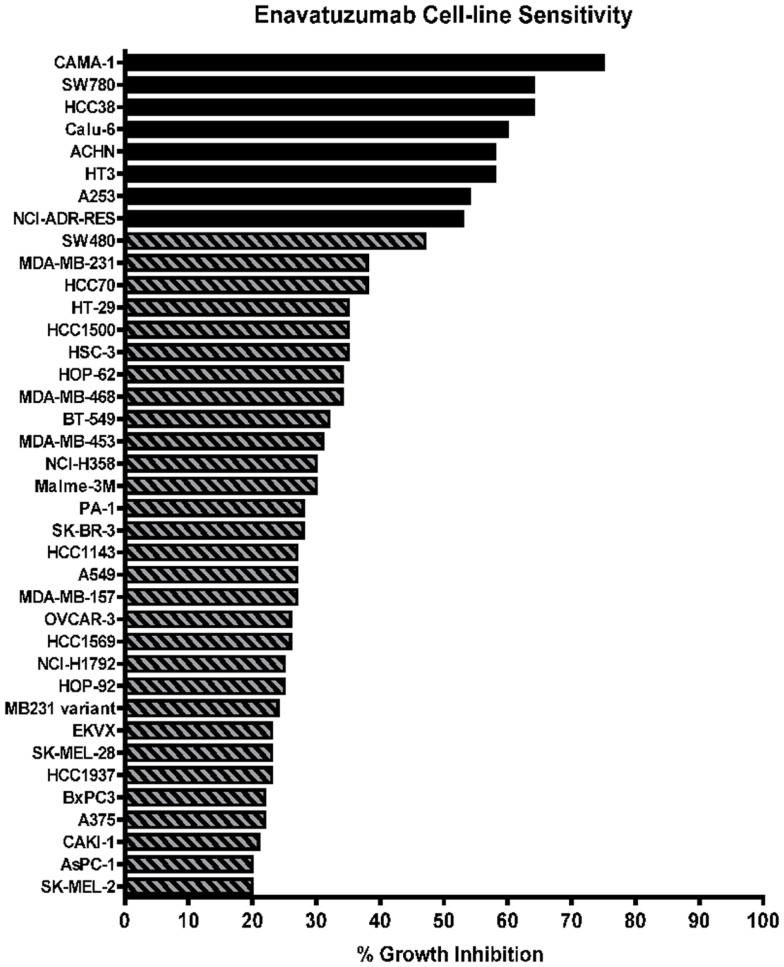
**Enavatuzumab has growth inhibitory activity in a broad range of cancer cell lines *in vitro***. One hundred and five cell lines representing multiple solid tumor types were tested for sensitivity to enavatuzumab in the presence of anti-human crosslinking antibody. The 38 cell lines that had ≥20% growth inhibition after 5-day treatment with enavatuzumab are shown.

A panel of 105 cancer cell lines representing the majority of solid tumor types was tested with enavatuzumab for *in vitro* growth inhibitory activity in a 5-day proliferation assay, in the presence of an anti-human F(ab′)_2_ to provide crosslinking of enavatuzumab (Figure 1). Cell surface TweakR expression was confirmed in 103 out of 105 cell lines by flow cytometry (data not shown). Of the 105 cell lines tested, enavatuzumab displayed ≥20% growth inhibitory activity in 38 of the cell lines (37%). Of these cell lines, 29 (28%) had ≥25% growth inhibition compared to cells treated with an isotype control antibody. Sixty-five TweakR-expressing cell lines were not sensitive to enavatuzumab, as indicated in Table 1. Enavatuzumab displayed activity in a broad range of tumor types with no single disease indication having notably superior sensitivity. However, two of the small cell lung cancer lines tested, NCI-H69 and NCI-H146 did not express TweakR and were not sensitive to enavatuzumab, demonstrating target specific activity.

**Table 1:** **Enavatuzumab sensitivity did not correlate with TweakR expression**.

Sensitive lines		Resistant lines			
Cell line	Expression	Cell line	Expression	Cell line	Expression
CAMA-1	5.8	786-0	7.5	NCI-H187	3.9
SW780	16.1	A2058	3.8	NCI-H209	2.4
HCC38	10.7	A498	9.4	NCI-H226	4.8
Calu-6	14.2	BT20	5.3	NCI-H23	8.4
ACHN	9.8	BT-474	4.7	NCI-H292	12.0
HT3	15.2	BT-483	2.6	NCI-H322M	1.4
A253	11.6	C32	17.1	NCI-H345	2.4
NCI-ADR-RES	72.2	Calu-3	4.8	NCI-H460	3.7
SW480	55.0	COLO-205	1.9	NCI-H520	1.9
MDA-MB-231	4.8	DMS-79	2.1	NCI-H522	3.6
HCC70	2.1	DU-4475	3.9	NCI-H596	2.9
HT-29	18.5	HCC-1428	4.0	*NCI-H69*	1.2
HCC1500	5.2	HCC2998	5.4	NCI-H82	2.0
HSC-3	8.3	HCT-116	26.3	OVCAR4	15.9
HOP-62	10.3	HCT-15	16.0	OVCAR-5	1.4
MDA-MB-468	2.6	Hs 578T	5.1	OVCAR-8	55.3
BT549	5.6	HT1376	21.6	RXF393	6.2
MDA-MB-453	1.8	HT-144	5.0	SK-MEL-5	3.6
NCI-H358	11.6	IGROV-1	8.5	SKOV3	19.4
Malme-3M	11.4	KM12	2.2	SN12C	8.2
PA-1	17.4	LoVo	3.7	SR	1.7
SK-BR-3	4.2	LOXIMVI	11.3	SW48	8.9
HCC1143	10.2	M14	10.1	SW620	4.5
A549	16.4	MCF-7	11.3	SW626	9.2
NCI-H157	4.9	MDA-MB-157	6.3	SW948	12.8
OVCAR-3	9.2	MDA-MB-175-VII	2.3	T47D	4.7
HCC1569	3.9	MDA-MB-361	6.2	TK10	18.4
NCI-H1792	1.9	MDA-MB-435	4.7	U031	15.4
HOP-92	19.7	MDA-MB-435S	28.6	UACC-257	2.8
MB231 variant	19.8	MIA-PaCa-2	6.9	UACC62	11.0
EKVX	26.1	MX-1	11.9	WM-115	7.9
SK-MEL-28	14.2	NCI-H1155	2.0	ZR-75-1	3.1
HCC1937	5.3	*NCI-H146*	1.2	ZR-75-30	4.2
BxPC3	21.4	NCI-H1838	4.6		
A375	8.9				
CAKI-1	9.0				
AsPC-1	3.6				
SK-MEL-2	6.2				

*Sensitive lines (*n* = 38) were defined as exhibiting at least 20% growth inhibition, and are arranged in order of sensitivity to enavatuzumab (see Figure 1). Resistant lines showed <20% growth inhibition and included 65 TweakR-expressing lines and 2 non-expressing lines, italicized (NCI-H146 and NCI-H69). TweakR expression was measured by enavatuzumab binding by flow cytometry and is reported as the fold increase in mean fluorescence intensity versus control antibody stained cells*.

### NFκB pathway is activated in response to enavatuzumab treatment

**Figure 2:**
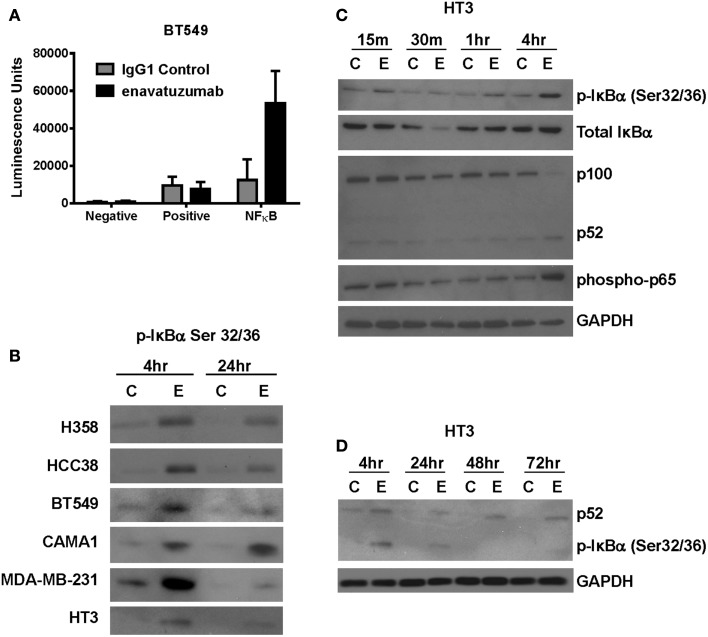
**The NFκB pathway is activated in response to enavatuzumab treatment *in vitro***. **(A)** A NFκB luciferase reporter construct was transfected into BT549 cells, then treated with antibody/crosslinker. Luciferase expression was measured 24 h post antibody treatment. **(B)** NFκB activation was examined by Western blotting for p-IκBα expression. Cell lysates were prepared at 4 and 24 h post antibody treatment. C, samples treated with control antibody/crosslinker; E, enavatuzumab/crosslinker-treated samples. **(C,D)** Enavatuzumab-induced the NFκB pathway in HT3 cells. HT3 cells were treated with enavatuzumab (E) or IgG1 control antibody (C) in the presence of crosslinking antibody for the times indicated. Cell lysates were prepared and activation of NFκB pathway members was analyzed by Western blot.

Multiple signaling pathways have been shown to be activated downstream of TweakR, including NFκB, ERK, and JNK. NFκB, in particular, has been suggested to play a pivotal role in the context of tumor cell growth, as TWEAK upregulates the NFκB signaling pathway in glioma cells where TWEAK also mediates resistance to cytotoxic agents (22). To determine if NFκB signaling might also be upregulated by TweakR agonists that inhibit tumor growth, we transfected enavatuzumab sensitive cells with a NFκB luciferase transcriptional reporter construct. Upon enavatuzumab treatment, we observed a marked increase in NFκB-driven luciferase expression in several cell lines, BT549 (Figure 2A), HCC38, A375, H358, and HT3 (data not shown). To investigate further the activation of the NFκB pathway by enavatuzumab, we next assessed the levels of phosphorylation of IκBα, the cytoplasmic inhibitor of classical NFκB subunits. Phosphorylation of IκBα on Serine 32/36 has been shown to reflect NFκB pathway activation, as it allows the cytoplasmic release and migration of NFκB dimers into the nucleus to initiate transcription (18, 23, 24). In enavatuzumab sensitive cell lines, we consistently observed phosphorylation of IκBα following enavatuzumab treatment (Figure 2B). Degradation of IκBα was observed 30 min post enavatuzumab treatment, but this degradation was transient, with IκBα expression being quickly re-established (Figure 2C). The phosphorylation of IκBα was observed at later time points 4 and 24 h, indicating sustained activation of the NFκB pathway, but this phosphorylation was not maintained at 48 and 72 h timepoints (Figure 2D). This phosphorylation event was seen in all sensitive cell lines tested, with no marked change in total IκBα expression at these time points (data not shown). In HT3 cells, cleavage of p100 to the activated subunit p52 was observed at 4 h and this activation was prolonged out to 72 h (Figures 2C,D).

### Enavatuzumab induces elevated NFκB subunit activation and increased downstream transcriptional changes in sensitive compared to resistant cell lines

**Figure 3:**
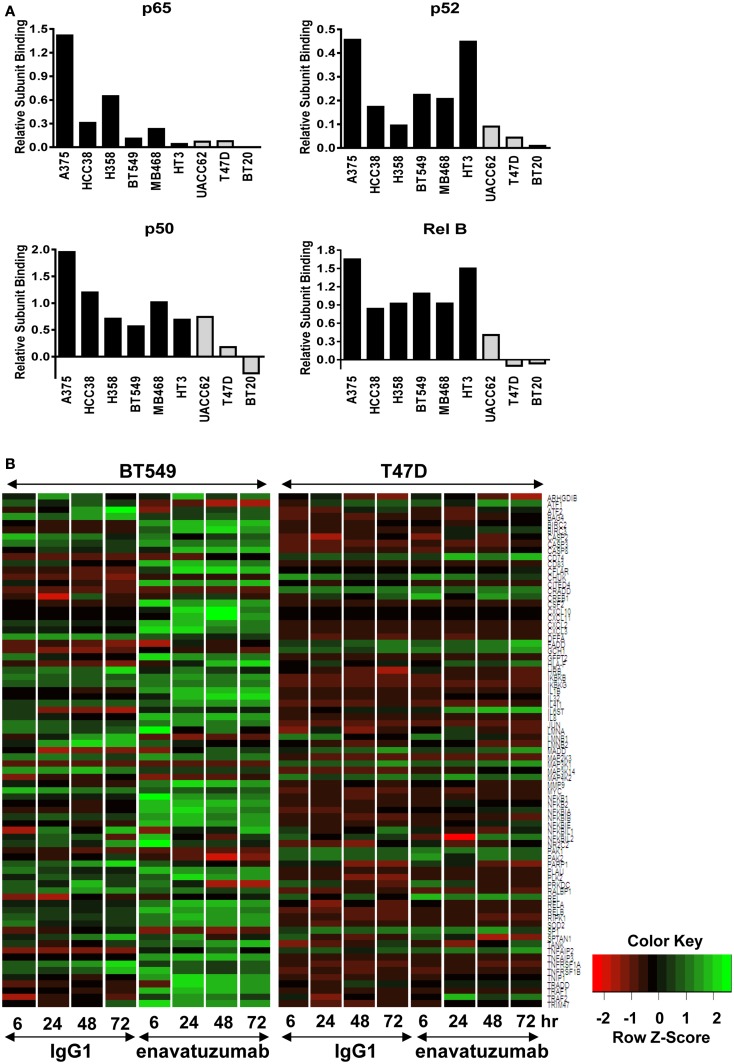
**Differential NFκB pathway activation in sensitive compared to resistant cell lines**. **(A)** NFκB functional DNA binding ELISAs were performed on sensitive (black bars) and resistant (gray bars) cell lines. The relative level of subunit binding (*y*-axis) was calculated by subtracting the OD_450_ of the control samples from that of the enavatuzumab-treated samples. **(B)** Enavatuzumab-induced changes in gene expression were measured 6–72 h after antibody treatment by gene expression profiling. The average of duplicate samples is shown for each time point treated with enavatuzumab or IgG1 control in the presence of a crosslinking antibody. Changes in NFκB responsive genes are shown for a sensitive (BT549) and resistant (T47D) cell line.

Having established that NFκB was activated in response to enavatuzumab treatment, we next performed NFκB functional ELISAs across multiple cell lines to determine which specific NFκB subunits were activated in response to enavatuzumab treatment. The NFκB DNA binding ability of six sensitive cell lines (A375, HCC38, H358, BT549, MDA-MB-468, and HT3) and three resistant cell lines (UACC62, T47D, BT20) were examined by ELISA post treatment (Figure 3A). Induction of all NFκB subunits (p50, p65, p52, RelB, and c-Rel) was observed in sensitive cell lines following treatment. This indicates that both classical (p50, p65) as well as non-classical (p52, RelB) NFκB pathways could be activated by enavatuzumab treatment. Interestingly, resistant cell lines showed significantly less induction of all NFκB subunits, with little or no subunit induction occurring in UACC62, T47D, and BT20.

The differential pattern of NFκB activation between enavatuzumab sensitive and resistant lines was further confirmed by gene chip microarray analysis. The number of genes up- or down-regulated >2-fold was greater in sensitive lines (BT549, MDA-MB-468) compared to those in resistant lines (T47D, BT20) (Table 2). For example, 408 genes were up- or down-regulated in sensitive BT549 cells after 24 h of treatment, while the expression of only 9 genes changed in the resistant T47D cells at this timepoint. Many of the transcriptional changes were for genes known to be regulated by NFκB (Figure 3B). Known NFκB pathway members and NFκB-regulated genes, as defined by GSES, are induced by enavatuzumab in BT549 cells. In contrast, few NFκB-regulated genes were induced in the T47D resistant cell line. Upregulation of NFκB-regulated genes was seen across all the time points in the BT549 gene chip, while for T47D, activation of the few NFκB-regulated genes appeared to be more delayed, and were often different genes than those in the sensitive lines.

**Table 2:** **Enavatuzumab treatment induced more gene expression changes in sensitive cell lines than in resistant lines**.

	Number of genes up or down >2-fold			
	Sensitive lines		Resistant lines	
	MDA-MB-468	BT549	T47D	BT20
6 h	51	126	1	nt
24 h	50	408	9	nt
48 h	329	648	15	3
72 h	445	766	13	nt

*The total number of genes whose expression is altered more than twofold up or down following enavatuzumab treatment at each time point is displayed*.

*nt, not tested. Analysis of BT20 cells was performed only at 48 h*.

### NFκB activation is seen *in vivo* following enavatuzumab treatment

**Figure 4:**
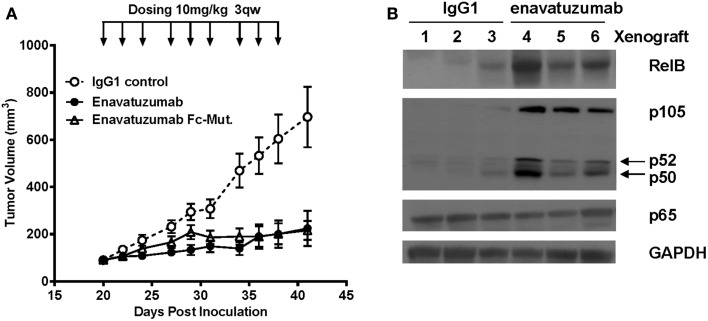
***In vivo* efficacy and NFκB activation in H358 xenografts in response to enavatuzumab**. **(A)** H358 xenograft tumors at ~100 mm^3^ in ICR SCID mice were treated i.p. with enavatuzumab, enavatuzumab-Fc mutant, or IgG1 control at 10 mg/kg q3w as indicated. **(B)** H358 xenografts were treated on days 0 and 3 with enavatuzumab or control antibody. Tumors from three mice from each treatment group were excised on day 4, after which cell lysates were prepared and analyzed by Western blot. Activation of NFκB was examined by measuring RelB, p105/p50, p52, and p65 levels in the tumors.

To determine whether NFκB was activated *in vivo* in response to enavatuzumab treatment, we selected the enavatuzumab sensitive H358 xenograft model, as H358 cells displayed strong NFκB activation and growth inhibition by enavatuzumab *in vitro*. In addition, enavatuzumab exhibited 70% tumor growth inhibition (TGI) of H358 tumors *in vivo*. In this model, response to enavatuzumab was dependent on signaling through TweakR, as a version of enavatuzumab containing a mutation in the Fc region that prevents antibody-dependent cellular cytotoxicity, exhibited equivalent activity as wild-type enavatuzumab (Figure 4A). Other sensitive lines, including HT3 and BT549, did not form xenografts in our hands. In H358 tumors harvested from enavatuzumab-treated mice, an increase in the levels of NFκB subunits RelB, p105/p50, and p52 was observed (Figure 4B), indicating activation of the NFκB pathway. No change in p65 expression was observed following enavatuzumab treatment in this model.

### Inhibition of NFκB pathway members reduces enavatuzumab-driven cancer growth inhibition

**Figure 5:**
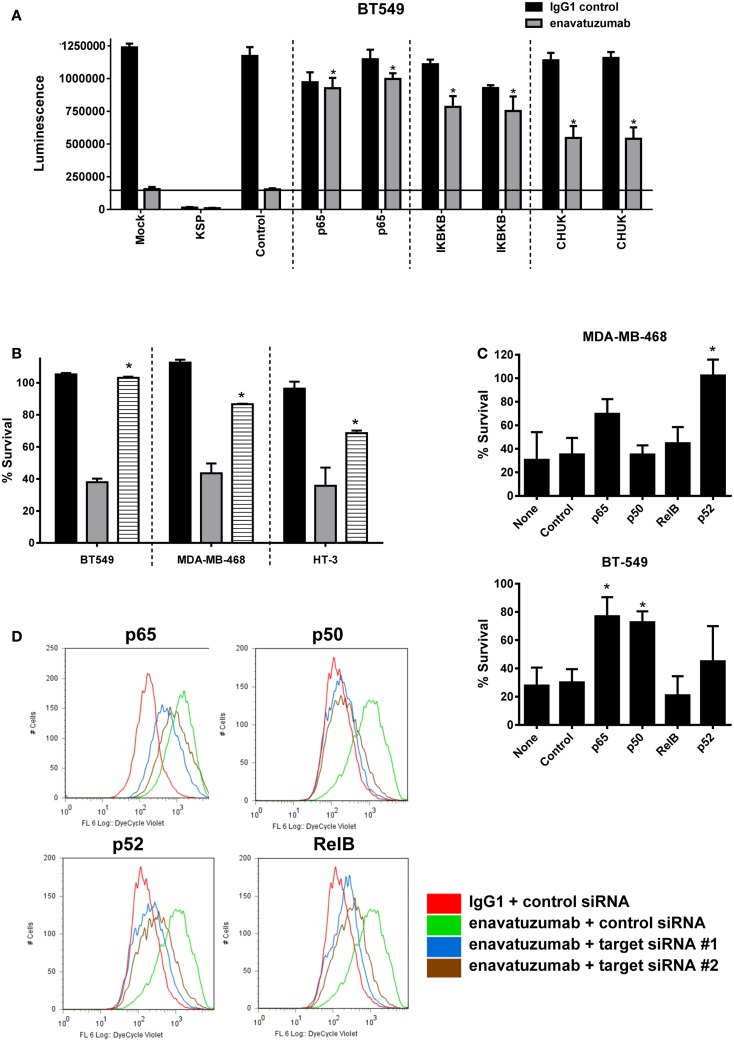
**Inhibition of NFκB activation prevents the growth inhibitory activity of enavatuzumab**. **(A)** BT549 cells were transfected with two different siRNAs against p65 or upstream kinases IKK1 (IκBKβ), IKK2 (CHUK). Transfection controls were non-targeting control (negative) or KSP (positive control for transfection efficiency). After 48 h, cells were treated with enavatuzumab or IgG1 control at 10 μg/mL for an additional 5 days in the presence of anti-human crosslinking antibody (3.5 μg/mL), and the cell viability was determined. siRNA significantly reduced growth inhibition by enavatuzumab, compared to mock or control siRNA transfected cells (horizontal line) (*p-value < 0.05). **(B)** Enavatuzumab sensitive lines were treated with IKK inhibitor IKK16 at 160 nM (black bars), enavatuzumab/crosslinker (gray bars), or IKK16 plus enavatuzumab/crosslinker (striped bars) and % survival was measured after 5 days. IKK16 significantly blocked growth inhibition by enavatuzumab compared to enavatuzumab alone (*p-value < 0.05). **(C)** Targeting individual NFκB subunits (p50, p65, RelB, p52) by siRNA reduced enavatuzumab activity in sensitive cell lines (MDA-MB-468 and BT549). Cells were transfected with pooled targeting siRNA or control siRNA for 48 h, prior to treatment with enavatuzumab or IgG1 control for 5 days in the presence of a crosslinking antibody. Percent survival was calculated from the relative viability of cells treated with enavatuzumab versus control-treated cells (*p-value < 0.05). **(D)** The effect of NFκB induced by enavatuzumab on cell division was investigated by pre-labeling HT3 cells with the Cell Trace™ reagent. Cells treated with enavatuzumab/crosslinker (green line) are compared to the IgG1 control-treated cells (red line). Cells transfected with two different siRNAs to p65, p50, p52, or RelB and treated with enavatuzumab/crosslinker (blue and brown lines) are also displayed.

Having shown that enavatuzumab treatment upregulates NFκB pathway activation, we next assessed whether NFκB signaling was important for enavatuzumab’s growth inhibitory activity. siRNA experiments were performed to knock down components of the NFκB pathway, after which cells were assessed for sensitivity to enavatuzumab. siRNAs to multiple NFκB pathway members, including the upstream kinases IKKα (CHUK) or IKKβ significantly reduced the sensitivity of cell lines to enavatuzumab (Figure 5A). To confirm the role of the NFκB pathway in growth inhibition by enavatuzumab, a small molecule inhibitor (IKK16) of the NFκB upstream kinases IKKα/β was also found to reduce growth inhibition by enavatuzumab in multiple cell lines (Figure 5B). Assessing the impact of siRNA knockdown of individual NFκB subunits (p65, p52, RelB, and p50) showed a differential reduction in growth inhibition by enavatuzumab. siRNA inhibition of p52 expression reduced enavatuzumab growth inhibition in MDA-MB-468 cells, while BT549 cells primarily showed a dependency on p50 and p65 for enavatuzumab activity (Figure 5C). Knockdown of RelB was found to reduce enavatuzumab growth inhibition in other cell lines, including HT3 (data not shown). The importance of each particular subunit for the observed growth inhibition varied between cell lines and did not necessarily correlate with induction of subunit expression or activation by enavatuzumab (Figures 3A,B).

We next investigated the mechanism by which enavatuzumab inhibits tumor cell growth inhibition through NFκB. The rate of cell proliferation in enavatuzumab-treated cells was assessed using CellTrace™ dye. The dye contained within the labeled cells is diluted when passed to daughter cells during cell division, resulting in reduced fluorescence in the progeny. HT3 cells treated with enavatuzumab exhibited higher fluorescence than control-treated cells, suggesting that they divided less frequently (Figure 5D). Inhibition of cell division by enavatuzumab was shown to be NFκB-dependent by using siRNAs against individual NFκB subunits (p50, p65, RelB, p52). Knockdown of each of these NFκB subunits allowed HT3 cells to divide more frequently, thereby overcoming inhibition of cell division by enavatuzumab.

### Enavatuzumab causes an NFκB-dependent upregulation of the cell cycle inhibitor p21

**Figure 6:**
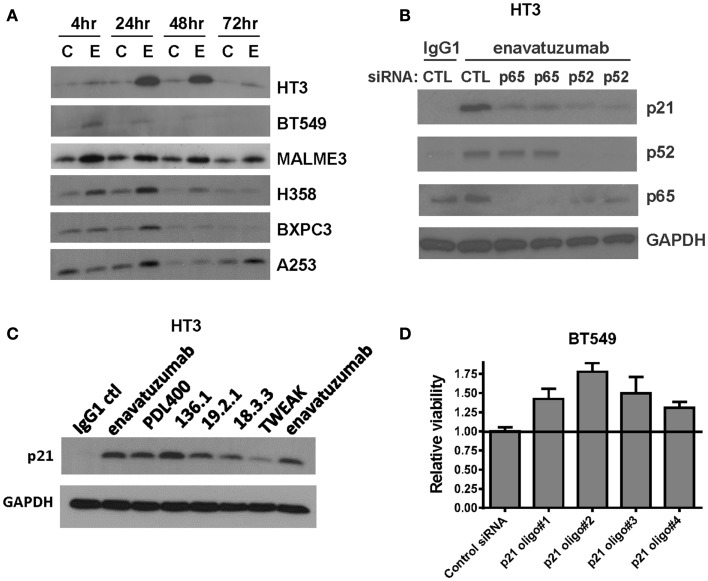
**Enavatuzumab inhibits cell division and upregulates p21 *in vitro* and *in vivo* in an NFκB-dependent manner**. **(A)** Sensitive cell lines HT3, BT549, MALME-3M, H358, BXPC3, and A253 were treated with IgG1 control antibody (C) or enavatuzumab (E) for 4–72 h in the presence of crosslinking antibody, after which p21 expression was assessed by Western blot. **(B)** The role of NFκB in enavatuzumab-induced p21 expression was examined by knocking down the expression of p65 or p52 with two different siRNAs to each. Samples were transfected with siRNA for 24 h, then treated with IgG1 control or enavatuzumab for an additional 24 h in the presence of crosslinking antibody. Cell lysates were analyzed by Western blot for p21, p52, and p65 expression. **(C)** p21 is activated by multiple antibodies targeting TweakR targeting and TWEAK ligand. HT3 cells were treated (10 μg/mL) with antibodies that bind and activate TweakR including enavatuzumab, PDL400, 136.1, 19.2.1, 18.3.3, or TWEAK ligand (300 ng/mL) for 24 h in the presence of crosslinking antibody. p21 expression was then assessed by Western blot. **(D)** siRNA inhibition of p21 reduces the relative growth inhibition caused by enavatuzumab treatment. BT549 cells were transfected with four different siRNA oligos targeting p21. Two days post transfection, cells were treated with enavatuzumab for 5 days in the presence of crosslinking antibody and cell viability was determined. Relative viability of 1.0 represents the viability of cells transfected with the non-targeting control siRNA and treated with enavatuzumab. An increase above 1.0 in cells transfected with p21 siRNAs indicates an increase in cell viability relative to the control siRNA transfected cells (*p* < 0.05). Control transfected cells treated with the IgG1 control antibody exhibited a relative viability of 3.75.

To characterize further the mechanism of cell growth inhibition by enavatuzumab, we next assessed whether inhibition of cell division might be achieved by altering cell cycle regulators. One well characterized NFκB-regulated cell cycle inhibitor is p21 (WAF1/CIP1) (25, 26). The expression of p21 was found to be upregulated in multiple sensitive cell lines after enavatuzumab treatment (Figure 6A). To confirm that p21 upregulation by enavatuzumab was mediated by NFκB, siRNAs against p65 and p52 were transfected into HT3 cells, after which they were treated with enavatuzumab. While siRNAs to both p65 and p52 downmodulated expression of their cognate proteins, they had little/no effect on the expression of the other subunit (Figure 6B). However, p21 upregulation by enavatuzumab was blocked by siRNAs to both p65 and p52, suggesting that p21 induction was NFκB-dependent. The ability of other TweakR agonists to effect p21 upregulation was also evaluated. Multiple TweakR agonists, including the ligand, TWEAK, and TweakR targeting antibodies with differing signaling potentials, including weak (PDL400, 18.3.3), moderate (19.2.1 and enavatuzumab), and strong (136.1) agonists, were all able to upregulate p21 in HT3 cells (Figure 6C), suggesting that TweakR stimulation by a variety of agonists activates common downstream signaling pathways.

To determine whether p21 upregulation was important for growth inhibition mediated by enavatuzumab, BT549 cells transfected with p21 siRNAs were treated with enavatuzumab. Knockdown of p21 expression reduced the ability of enavatuzumab to inhibit the growth of BT549 cells by ~20% (Figure 6D). Taken together, these results suggest that growth inhibition by TweakR agonists is mediated by p21 upregulation, which is, in turn, driven by NFκB pathway activation.

**Figure 7:**
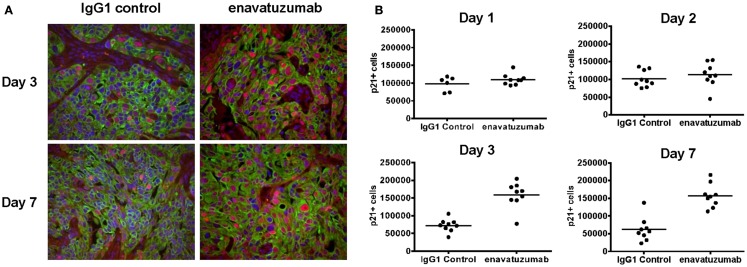
**Enavatuzumab treatment of H358 xenograft tumors results in p21 upregulation *in vivo***. **(A)** H358 xenografts were grown in ICR SCID mice to ~100 mm^3^ and mice were then treated with a single i.p. injection of IgG1 control or enavatuzumab at 10 mg/kg. Tumors were excised and the levels of p21 were determined by multi-color IHC. A representative image with p21 expression in red, cytokeratin in green, and nuclei in blue is shown (40× magnification). **(B)** Quantification of p21 expression from H358 xenografts excised on days 1, 2, 3, and 7 post treatment. Data points represent a single 40× magnification field with three fields for three individual xenografts analyzed per time point for each treatment (*p* < 0.001 IgG1 control versus enavatuzumab days 3 and 7). The *y*-axis represents quantification of p21+ cells in arbitrary units.

Having shown that enavatuzumab-mediated growth inhibition by upregulating p21 *in vitro*, enavatuzumab was next evaluated for its ability to induce p21 *in vivo*. H358 xenografts were treated with a single dose of enavatuzumab, after which tumors were harvested and stained for p21 by immunohistochemistry (Figure 7A). Enavatuzumab treatment resulted in an increase in p21-positive cells that was observed 3 days after dosing, and was maintained for at least 1 week after a single i.p. dose of enavatuzumab (Figure 7B).

## Discussion

In this study we have demonstrated that enavatuzumab caused a growth inhibitory effect on many cancer cell lines, across a range of tumor types. This agrees with the broad *in vivo* activity seen in xenograft models (17) and supports the investigation of enavatuzumab as a therapeutic modality in solid tumors. Growth inhibition and signaling was enhanced through the crosslinking of enavatuzumab. This implies that TweakR oligomerization is important for enavatuzumab-mediated growth inhibition and for the signaling that occurs downstream of the receptor. This agrees with previous reports assessing TweakR activation with agonist antibodies (15, 27).

It should be noted that the extent of cell surface expression of TweakR did not correlate with *in vitro* activity, as discussed previously (15, 17). Therefore the highest TweakR-expressing cell lines were not necessarily the most enavatuzumab sensitive. This is likely due to the pleiotropic functions of TweakR and reveals a degree of complexity regarding the relationship between receptor expression and function that requires further investigation.

The induction of NFκB in response to enavatuzumab stimulation of TweakR agrees with findings in the literature for the receptor’s natural ligand TWEAK (2, 9, 28). Like TWEAK, enavatuzumab activated both classical and non-classical NFκB pathways. Previous reports have demonstrated transient activation of p50/p65 NFκB subunits and a more sustained activation of the non-classical p52/RelB subunits in response to TWEAK (29, 30). Interestingly we observed phosphorylation of the NFκB inhibitor IκBα up to 24 h post enavatuzumab treatment. This indicates prolonged activation and continual re-expression of IκBα despite NFκB activation. This contrasts with what is observed with TNFα, where IκBα is rapidly phosphorylated and degraded leading to transient activation of NFκB (31). We also saw activation of p52 up to 72 h post addition of enavatuzumab. The finding that NFκB pathway induction was observed several days post enavatuzumab treatment by Western blot and microarray analyses indicates some possible differential activity between that of TweakR’s ligand TWEAK and enavatuzumab.

Transient versus persistent NFκB activation can result in different gene induction profiles in response to TNFα and LPS, resulting in contrasting protein expression and varying growth and survival phenotypes (32, 33). Therefore continual signaling through TweakR by enavatuzumab may induce different NFκB-regulated genes than when TWEAK binds TweakR. Also, it has been previously reported that TWEAK can be internalized (10, 34), and like other TNF family members, the cell surface expression of the receptor TweakR is likely to be tightly regulated/internalized upon ligand stimulation (35, 36). In contrast, antibody binding to TweakR maintains cell surface expression of TweakR (data not shown), which may lead to the observed prolonged receptor activation and sustained stimulation of downstream signaling pathways, such as NFκB. Alternatively, the crosslinking of enavatuzumab may aid in prolonging the signaling downstream of TweakR which could explain differences in persistence of signaling seen with TWEAK ligand versus enavatuzumab. Such a relationship between antibody crosslinking and downstream signaling has been described previously (37).

Cell lines sensitive to enavatuzumab displayed more NFκB activation, as seen by subunit functional ELISA, compared to resistant cell lines. Resistant cell lines also showed a delayed and reduced induction of NFκB-regulated genes. Gene expression analysis revealed strong induction of many NFκB-regulated genes across all time points tested (6–72 h), while T47D cells had little induction of the same NFκB-regulated genes, especially at the earlier time points. Sensitive cell lines showed 400–700 genes up or down-regulated >2-fold following enavatuzumab treatment, while resistant cells exhibited changes in expression in fewer than 20 genes, indicating that significant transcriptional activity occurred in sensitive cell lines, which was not observable in the resistant setting. A small number of NFκB-regulated genes were induced in the T47D cell line, which were not induced in BT549 cells, which may suggest that some of these genes may play a role in the resistant phenotype, but their role, if any, is unclear. In general, it appears that sensitive cell lines exhibit increased overall activation of NFκB signaling by enavatuzumab, and these changes could potentially be useful when selecting patients and measuring their response. Therefore, understanding the molecular changes downstream of TweakR signaling in the sensitive/resistant settings is of great interest.

We demonstrated that the NFκB activation observed in response to enavatuzumab was essential for its growth inhibitory activity. siRNA targeting of specific NFκB subunits (p50, p65, p52, RelB) abrogated the growth inhibition, however different cell lines were more sensitive to inhibition of certain subunits than others. For example, HT3 cells were dependent on non-classical (p52/Rel B) NFκB activation, while BT549 cells relied more on the classical NFκB (p50/p65) pathway for enavatuzumab growth inhibition. All sensitive cell lines showed a marked increase in the expression of NFκB subunits by enavatuzumab, but there was no clear pattern of subunit induction in response to enavatuzumab treatment, and the induction did not always correlate with siRNA subunit sensitivity. It should be noted however, that we were able to reduce enavatuzumab activity in every sensitive cell line by inhibiting at least one or more NFκB subunits. Therefore, there does not appear to be a single reliance on either the classical or non-classical NFκB pathways for enavatuzumab activity, but is likely to comprise a complex combination of cellular events involving cross-talk and interdependency between both pathways that requires further examination.

Enavatuzumab-induced growth inhibition of many cancer cell lines, but the characteristics of this growth inhibition had not previously been described. We found that enavatuzumab treatment resulted in a marked reduction in cell division. In HT3 cells, siRNA inhibition of p50, p52, and RelB in the context of enavatuzumab treatment helped restore the number of cell divisions to that seen with IgG1 control. This further confirmed NFκB’s critical role in enavatuzumab’s growth inhibitory activity.

When we evaluated the possible causes of the cell division block caused by enavatuzumab, we found that the key cell cycle regulator p21 was induced following treatment in many responder cell lines both *in vitro* and in H358 xenografts *in vivo*. The prolonged *in vivo* upregulation of the cell cycle inhibitor p21, up to 7 days after a single antibody dose, is a compelling growth inhibitory mechanism of enavatuzumab and is a novel activity for a TweakR-targeted therapeutic. p21 was found to be upregulated by multiple TweakR-targeted molecules that have different levels of agonism, suggesting a common mechanism of growth inhibition by diverse agents that activate the TweakR signaling pathway. We were able to block enavatuzumab-driven p21 upregulation by inhibiting specific NFκB subunits. Inhibition of p52 in particular, resulted in complete loss of p21 induction in HT3 cells, further supporting the importance of the non-classical NFκB pathway for enavatuzumab’s activity in this cell line.

It has been reported that TweakR activation can result in growth inhibition by NFκB-mediated upregulation of TNF and subsequent apoptosis via TNFR1 in some cell lines (e.g., Kym-1, OVCAR4, SKOV3) (38, 39). Interestingly, two of these cell lines, OVCAR4 and SKOV3 were included in our screen and were found not to be sensitive to enavatuzumab. However, because not all enavatuzumab sensitive cell lines were assessed for p21 upregulation, it is possible that additional mechanisms, including TNF-mediated apoptosis, may be involved in the growth inhibition by enavatuzumab. This question warrants further evaluation.

The NFκB-dependent growth inhibitory activity of enavatuzumab is an interesting finding for a family of transcription factors frequently associated with growth and survival in cancer (40–42). However, the diversity and complexity of NFκB signaling suggests that NFκB can inhibit the growth of tumor cells under certain circumstances (43).

Therefore, our finding that the growth inhibitory activity of enavatuzumab is driven in large part by NFκB suggests that the targeted activation of the NFκB pathway may be a novel therapeutic approach for treating cancer. In summary we have outlined how NFκB activation and induction of downstream signaling events, including p21, are essential for the TGI mediated by enavatuzumab.

## Author Contributions

All authors are employes or former employes of AbbVie Biotherapeutics Inc., or Abbott Biotherapeutics. The design, study conduct, and financial support for this research was provided by AbbVie. James W. Purcell and Han K. Kim participated in the design and execution of experiments; interpretation of results; and drafted, revised, and approved the final manuscript. Sonia G. Tanlimco and Minhtam Doan participated in the design and execution of experiments, interpretation of results; and revised and approved the final manuscript. Melvin Fox participated in the execution of experiments, interpretation of results; and revised and approved the final manuscript. Peter Lambert participated in the interpretation of results; and revised and approved the final manuscript. Debra T. Chao participated in the design and execution of experiments, interpretation of results; and revised and approved the final manuscript. Mien Sho participated in the design and execution of experiments; and revised and approved the final manuscript. Keith E. Wilson participated in the design of experiments; interpretation of results; and revised and approved the final manuscript. Gary C. Starling participated in the design of experiments; interpretation of results; and revised and approved the final manuscript. Patricia A. Culp participated in the design of experiments; interpretation of results; and revised and approved the final manuscript. AbbVie participated in the review and approval of the manuscript.

## Conflict of Interest Statement

All authors are current or former employees of AbbVie Biotherapeutics Inc., or Abbott Biotherapeutics Corp.
